# Gut Microbiota Comparison in Rectal Swabs Versus Stool Samples in Cats with Kidney Stones

**DOI:** 10.3390/microorganisms12122411

**Published:** 2024-11-24

**Authors:** Patrick Joubran, Françoise A. Roux, Matteo Serino, Jack-Yves Deschamps

**Affiliations:** 1 NP3, Nutrition, PathoPhysiology and Pharmacology Unit, Oniris VetAgro Bio, Nantes-Atlantic College of Veterinary Medicine, Food Science and Engineering, La Chantrerie, CEDEX 03, 44307 Nantes, France; patrick.joubran@oniris-nantes.fr (P.J.); francoise.roux@oniris-nantes.fr (F.A.R.); 2 Emergency and Critical Care Unit, Oniris VetAgro Bio, Nantes-Atlantic College of Veterinary Medicine, Food Science and Engineering, La Chantrerie, CEDEX 03, 44307 Nantes, France; 3 IRSD, Institut de Recherche en Santé Digestive, Institut National de la Santé et de la Recherche Médicale (INSERM) U1220, Institut National de Recherche pour l’Agriculture, l’Alimentation et l’Environnement (INRAE), Ecole Nationale Vétérinaire de Toulouse (ENVT), Université de Toulouse III-Paul Sabatier (UPS), CS 60039, 31024 Toulouse, France; matteo.serino@inserm.fr

**Keywords:** microbiota, kidney stones, renal calculi, lithiasis, calcium oxalate, cat

## Abstract

To investigate the role of the intestinal bacterial microbiota in the pathogenesis of calcium oxalate nephrolithiasis in cats, a condition characterized by the formation of kidney stones, it is desirable to identify a sample collection method that accurately reflects the microbiota’s composition. The objective of this study was to evaluate the impact of fecal sample collection methods on the intestinal microbiota composition in two cat populations: healthy cats and kidney stone-diseased cats. The study included eighteen cats from the same colony, comprising nine healthy cats and nine cats with spontaneously occurring presumed calcium oxalate kidney stones. Three fecal collection methods were compared: rectal swabs, the collection of fresh stool, and the collection of stool exposed to ambient air for 24 h. The bacterial microbiota was analyzed through the high-resolution sequencing of the V3–V4 region of the 16S rRNA gene. For all cats, within the same individual, a one-way PERMANOVA analysis showed a significant difference between the rectal swabs and fresh stool (*p* = 0.0003), as well as between the rectal swabs and stool exposed to ambient air for 24 h (*p* = 0.0003), but no significant difference was identified between the fresh stool and non-fresh stool (*p* = 0.0651). When comparing the two populations of cats, this study provides seemingly conflicting results. (1) A principal component analysis (PCA) comparison revealed a significant difference in the bacterial composition between the healthy cats and the cats with kidney stones only when the sample was a fresh fecal sample (*p* = 0.0037). This finding suggests that the intestinal bacteria involved in the pathogenesis of kidney stones in cats are luminal and strictly anaerobic bacteria. Consequently, exposure to ambient air results in a loss of information, preventing the identification of dysbiosis. For clinical studies, non-fresh stool samples provided by owners does not appear suitable for studying the gut microbiota of cats with kidney stones; fresh stool should be favored. (2) Interestingly, the rectal swabs alone highlighted significant differences in the proportion of major phyla between the two populations. These findings highlight the critical importance of carefully selecting fecal collection methods when studying feline gut microbiota. Combining rectal swabs and fresh stool sampling provides complementary insights, offering the most accurate understanding of the gut microbiota composition in the context of feline kidney stone pathogenesis.

## 1. Introduction

The microbiota refers to the community of microorganisms (bacteria, archaea, fungi, protozoa, and viruses) that live within a living organism. The microbiota acts in symbiosis with its host and contributes to its health by performing various functions, notably digestive, metabolic, and immune functions. In humans, disturbances in the composition of the intestinal microbiota, referred to as “dysbiosis”, are suspected to be involved in the pathogenesis of diseases that do not appear to have a direct link with the intestine, including obesity, diabetes, hypertension, Alzheimer’s disease, autism, etc. [1]; these diseases have seen a marked increase in recent decades. Calcium oxalate nephrolithiasis, a condition characterized by the formation of kidney stones, has also increased significantly in recent years, and for this disease as well, the involvement of the intestinal microbiota is suspected. A dysbiosis could affect the intestinal degradation of oxalate, which could increase intestinal oxalate absorption and lead to hyperoxaluria and, consequently, increase the risk of calcium oxalate stone formation in the kidney [2,3,4,5,6,7,8].

In cats, kidney stones are almost exclusively (98%) composed of calcium oxalate [9,10]. Calcium oxalate kidney stones occur primarily in two forms: monohydrate (whewellite) and dihydrate (weddellite) forms. Calcium oxalate monohydrate stones are generally associated with hyperoxaluria, whereas calcium oxalate dihydrate stones are more commonly linked to hypercalciuria. In cats, calcium oxalate stones are predominantly of the monohydrate type. In the study by Kopecny et al. [10], out of 1820 calcium oxalate stones analyzed in cats, only 0.9% were pure dihydrate stones, 56.6% were monohydrate stones, and 42.6% were mixed stones containing both calcium oxalate monohydrate and dihydrate stones. Thus, nearly 99% of kidney stones in cats contain calcium oxalate monohydrate. These findings suggest that hyperoxaluria is likely the predominant mechanism leading to kidney stone formation in this species.

As in humans, nephrolithiasis in cats has significantly increased in recent decades [10,11,12,13,14,15]. The involvement of intestinal dysbiosis in the pathogenesis of this emerging disease remained to be demonstrated, which is why, in the main study of this project, we compared the microbiota of cats with kidney stones to that of healthy cats; our research confirmed the existence of intestinal and urinary dysbiosis in the kidney stone-diseased cats [16]. Understanding the role of the intestinal microbiota in the formation of kidney stones in cats is of interest for the development of new therapeutic and preventive approaches based on the modulation of the intestinal microbiota [17,18]. The collection method can influence the composition of the identified bacterial microbiota; the oxygen gradient decreases from the intestinal mucosa to the intestinal lumen [19,20,21], so the bacteria of the intestinal mucosa are more tolerant to oxygen than the bacteria of the intestinal lumen. The bacterial populations colonizing the intestinal biofilm are therefore potentially different from those colonizing the intestinal lumen [21,22,23]. The analysis of a rectal swab would provide information on bacteria adherent to the intestinal mucosa, while stool analysis would provide information on luminal bacteria [24]. The use of an inappropriate sampling method could limit the detection of bacteria involved in feline kidney stone formation; if the responsible bacteria were primarily adherent to the mucosa, they would be less easily identifiable in stool samples, and conversely, if the involved bacteria were mainly luminal, they would be less detectable with a rectal swab. Prior to conducting research on the role of the intestinal microbiota in the pathogenesis of feline kidney stones, it is desirable to identify the collection method most likely to detect the bacteria involved.

Collecting spontaneously produced feces immediately after defecation is the easiest method to obtain a fecal sample from cats. Rectal swabs can be challenging with uncooperative cats; this method is uncomfortable for non-anesthetized animals, which increases the risk of contamination by bacteria from the skin or anal region, and preparing the perianal area aseptically can also be unpleasant for the cat. Thus, high-quality rectal swabs are primarily feasible in anesthetized cats, for instance, during an imaging procedure. In human medicine, rectal swabs are more commonly used for pathogen detection than for precise metagenomic analyses [25].

Whether it involves cats living in a cattery or cats living in private homes, it is not always easy to collect stool immediately after its production. Since the bacteria of the intestinal microbiota are mostly anaerobic [26,27], the analysis of stool exposed to ambient air for several hours may not reflect the microbiota composition with sufficient fidelity.

The first objective of this preliminary study was to compare the impact of three fecal sample collection methods on the composition of the intestinal bacterial microbiota of the same subject. The second objective of this study was to compare the composition of the intestinal bacterial microbiota in two populations of cats, healthy cats and kidney stone-diseased cats, to identify potential intestinal dysbiosis in the cats with renal calculi.

## 2. Materials and Methods

### 2.1. Studied Population

The cat population in this preliminary study has been described in detail in the main study [16]; only the essential elements are summarized here.

#### 2.1.1. Recruitment of Cats

The Nutrition Service of our institution (Oniris, National Veterinary School of Nantes, France) maintained a cattery housing about one hundred domestic cats (*Felis catus*) designated for studying the qualities of certain foods or dietary supplements. Among these cats, of all the domestic shorthair cats that were sterilized before puberty and were healthy at the age of one year, some spontaneously developed kidney stones in the years following their arrival, on average at about the age of 7 years. Cats with kidney failure and those experiencing pain were euthanized. For the present study, the remaining 9 asymptomatic neutered cats with renal lithiasis were enrolled. Additionally, an equivalent number of 9 control cats from the same colony, living under identical conditions and free from renal calculi, were enrolled to form a control group, making a total of 18 cats.

#### 2.1.2. Living Conditions

All cats were fed the same commercial senior dry food (Mature Consult Balance S/O, Royal Canin^®^, Aimargues, France) (see Supplementary Data for diet composition) for at least a year at a consistent daily ration, free fed collectively at a rate of 50 g/cat. The cats were housed in large aviaries containing 8 individuals that could interact freely; cats with renal lithiasis and healthy cats were not separated.

#### 2.1.3. Inclusion Criteria

The diagnosis of renal lithiasis or the absence of renal calculi was made using radiography and ultrasound. To be retained, cats with lithiasis had to present with one or more kidney stones visible by radiography in at least one kidney, and the presence of the stones had to be confirmed by ultrasound. Cats from the control group were considered healthy if no stone of the upper urinary tract was visible by radiography or ultrasound. Given the studied species (domestic cat), the location of the stones (in the upper urinary tract), the radiological density of the stones, and their small size, the observed stones were assumed to be calcium oxalate stones.

### 2.2. Collection of Fecal Samples

For each cat, three stool collection methods were studied: rectal swabs, the collection of fresh stool, and the collection of stool exposed to ambient air for 24 h. To ensure comparability between samples, the three collection techniques were performed on the same day, by the same individuals (the authors) and at the same site (the rectum). The cats fasted for 3 h in preparation for anesthesia. Prior to the anesthesia, abdominal palpation was performed to confirm the presence of stool in the rectum. If no stool was palpable, the sampling procedure was postponed. When stool was palpable in the rectum, the cats were anesthetized using a combination of medetomidine (20 µg/kg) and ketamine (7 mg/kg) administered intramuscularly and were then left to rest until the drugs took effect.

#### 2.2.1. Rectal Swab

Rectal swab samples were collected using a standard swab, without transport medium, inserted into the rectum through the anus, 2 cm from the anal margin. Once in contact with the rectal mucosa, the sample was collected by gently rotating the swab 360° for a few seconds, while avoiding, as much as possible, collecting any stool content. The swab was then placed back in its original sterile container and immediately frozen at −80 °C.

#### 2.2.2. Fresh and Non-Fresh Stool

Once the rectal swab was completed, a fresh stool sample was collected by gently inserting a sterile gloved middle finger into the rectum, thus mimicking a spontaneous defecation. Half of the collected stool was placed intact with sterile forceps in a sterile tube and immediately frozen at −80 °C to constitute the fresh stool sample. The other half of the stool was placed in a sterile tube left open to ambient air and room temperature for 24 h. After 24 h, the tube was closed and placed in a freezer at −80 °C to constitute the non-fresh stool sample. If no stool was accessible with the finger in the rectum, the entire procedure was repeated on another day.

### 2.3. Stool Analysis

Once all samples were collected, they were transported on dry ice in a single shipment to the laboratory for analysis. After thawing, the laboratory sampled the center of each piece of stool, thereby avoiding analysis of the stool periphery.

For the first part of the study, the intestinal microbiota composition was compared for each collection method from the same subject.

For the second part of the study, the intestinal microbiota composition was compared between the two populations: the healthy cats and kidney stone-diseased cats.

### 2.4. DNA Isolation and 16S rRNA Amplicon Sequencing

#### 2.4.1. High-Throughput Bacterial DNA Sequencing Techniques Used

Bacteria in the intestinal microbiota are largely uncultivable, which is why the microbiota is studied using metagenomics, a DNA sequencing technique [28]. In this study, 16S rRNA pyrosequencing was used to identify the bacterial composition of the microbiota, without resorting to global metagenomics. 16S rRNA is present only in bacteria; hence, only the bacterial microbiota was addressed, while archaea, viruses, fungi, and protists were not studied.

#### 2.4.2. DNA Extraction

DNA was extracted using the ZymoBIOMICS™ 96 MagBead DNA Kit (Zymo Research Corp., Tustin, CA, USA) following a protocol with dual cell lysis (mechanical and chemical). DNA isolation was carried out using a KingFisher Flex automated station (Thermo Fisher Scientific Inc., Boston, MA, USA) according to the manufacturer’s instructions. The DNA was quantified by fluorimetry using a Qubit^®^ 2.0 (Thermo Fisher Scientific).

#### 2.4.3. 16S rRNA Gene Amplification and Sequencing 

The V3–V4 region of the gene encoding the bacterial 16S ribosomal RNA was amplified by polymerase chain reaction (PCR) [29] using the primers 341F and 785R [30]. The amplicons were cleaned up using magnetic AMPure XP beads (Beckman Coulter, Villepinte, France) before adding dual indices and sequencing adapters using the Illumina Nextera XT Index kit (Illumina, San Diego, CA, USA). Each library was cleaned up and quantified by fluorimetry (Qubit^®^ 2.0 Fluorometer), normalized, and pooled. The pooled library was denatured before sequencing (2 × 250 paired-end, v2 chemistry) using an Illumina MiSeq Sequencing system (Illumina, San Diego, CA, USA).

#### 2.4.4. Data Processing

The sequences were analyzed using a bioinformatic pipeline developed by Biofortis based on Dadaist2 software [31]. After demultiplexing the indexed reads, single-read sequences were paired for each sample into longer fragments and cleaned. After quality filtering and sequencing error modeling, amplicon sequence variants (ASVs) were obtained. A taxonomic assignment of these ASVs was performed in order to determine bacterial community profiles.

### 2.5. Statistical Analyzes

Principal component analysis (PCA) graphs were generated, and diversity indices based on operational taxonomic units (OTUs) were calculated using the software PAST (PAleontological Statistics) version 4.10 [32]. A one-way PERMANOVA was used to determine whether the differences between methods were significant. Differences in the relative abundance of phyla were evaluated using a non-parametric multiple comparison test (a Wilcoxon test for each pair). Calculations were performed using the software PRISM version 5.0a. A *p*-value below 0.05 was considered statistically significant.

## 3. Results

### 3.1. Studied Population

This study enrolled nine cats with spontaneous renal lithiasis presumed to be calcium oxalate stones and nine healthy control cats. The group of cats with renal lithiasis included nine females, while the healthy cat group included seven females and two males. None of the cats in the study had ever experienced hormonal influence, as all the cats were sterilized before puberty. The age of the cats on the day of the procedure ranged from 9 to 10.8 years; the median age was 9.1 years. The median weight of the cats was 3.975 kg (±925 g).

The exact composition of the kidney stones was only identified for the three cats that died out of the nine cats with renal lithiasis during the main study performed the following year; as expected, for these three cats, the body of the stones consisted of 100% calcium oxalate monohydrate. The oxalocalcic nature of the other six cats’ stones was assumed on a probabilistic basis, knowing that 98% of upper urinary tract stones are calcium oxalate stones in cats [9,10] and given that struvite stones have a distinct radiological appearance: they are less radiodense, larger, smoother, and sometimes pyramid-shaped. Thus, there was a very low risk that one of the six cats had stones that were not calcium oxalate.

A total of 54 (18 × 3) fecal samples were collected from the 18 cats in the study. All 18 cats provided fecal samples that were deemed quantitatively sufficient for 16S rRNA analysis.

### 3.2. PCA According to the Collection Method and the Cat Population Studied

#### 3.2.1. A Comparison of the Three Stool Collection Methods in the Healthy Cats

**Figure 1:**
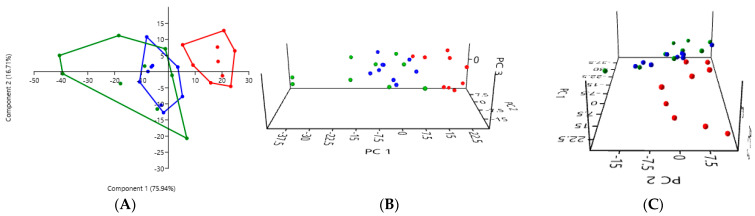
(**A**) 2D PCA graphs comparing the three collection methods in the healthy cats, with rectal swabs in red, fresh stool in blue, and non-fresh stool in green. (**B**,**C**) Exploring the same previously described patterns with 3D PCA plotting.

PCA comparing the three collection methods in the healthy cats revealed a clear difference between the analysis of the rectal swabs (in red) and the analysis of the stool, whether fresh (in blue) or exposed to ambient air for 24 h (in green) (Figure 1). The PCA graphs of the fresh stool and non-fresh stool overlap, suggesting that there were no significant differences between these two collection methods.

In the healthy cats, a one-way PERMANOVA analysis showed a significant difference between the rectal swabs and fresh stool (*p* = 0.0001), as well as between the rectal swabs and stool exposed to ambient air for 24 h (*p* = 0.0003). In contrast, no significant difference was observed between the fresh and non-fresh stool (*p* = 0.1927), indicating that both methods provided comparable results (Table 1).

#### 3.2.2. A Comparison of the Three Stool Collection Methods in the Kidney Stone-Diseased Cats

**Figure 2:**
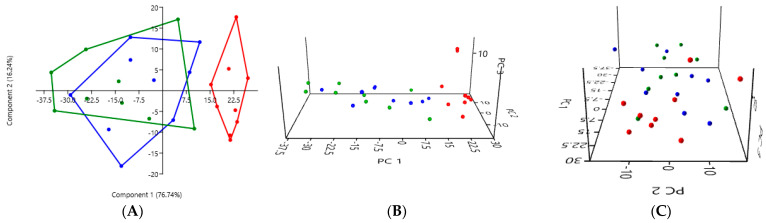
(**A**) 2D PCA graphs comparing the three collection methods in the kidney stone-diseased cats, with rectal swabs in red, fresh stool in blue, and non-fresh stool in green. (**B**,**C**) Exploring the same previously described patterns with 3D PCA plotting.

The PCA comparing the three collection methods in the kidney stone-diseased cats also revealed a clear difference between the analysis of the rectal swabs (in red) and the analysis of the stool, whether fresh (in blue) or exposed to ambient air for 24 h (in green) (Figure 2). The PCA graphs of the fresh stool and non-fresh stool overlap, suggesting that there was no significant difference between these two collection methods.

In the kidney stone-diseased cats, a one-way PERMANOVA analysis showed a significant difference between the rectal swabs and fresh stool (*p* = 0.0001), as well as between the rectal swabs and stool exposed to ambient air for 24 h (*p* = 0.0001). In contrast, no significant difference was observed between the fresh and non-fresh stool (*p* = 0.3634), indicating that both methods provided comparable results (Table 2).

#### 3.2.3. A Comparison of the Three Stool Collection Methods Among All Cats

**Figure 3:**
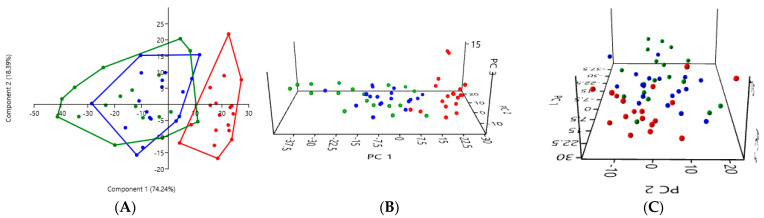
(**A**) 2D PCA graphs comparing all 18 cats with the three collection methods, with rectal swabs in red, fresh stool in blue, and non-fresh stool in green. (**B**,**C**) Exploring the same previously described patterns with 3D PCA plotting.

The PCA comparing the three collection methods among all eighteen cats, the nine healthy cats and the nine kidney stone-diseased cats confirmed a difference between the analysis of the rectal swabs (in red) and the analysis of the stool, whether fresh (in blue) or exposed to ambient air for 24 h (in green) (Figure 3). The PCA graphs for the fresh stool and non-fresh stool overlap, suggesting that there was no significant difference between these two collection methods.

Among all the cats, a one-way PERMANOVA analysis showed a significant difference between the rectal swabs and fresh stool (*p* = 0.0003), as well as between the rectal swabs and stool exposed to ambient air for 24 h (*p* = 0.0003). In contrast, no significant difference was observed between the fresh stool and non-fresh stool (*p* = 0.0651), indicating that the two methods provided comparable results (Table 3).

### 3.3. A Comparison of Main Bacterial Phyla According to the Method of Collection and the Studied Cat Population

To clarify the observed differences, bacterial phyla were studied for each collection method and within each population based on the relative abundance values obtained for each phylum. A Wilcoxon test was used to compare the methods two by two (rectal swab vs. fresh stool, rectal swab vs. non-fresh stool, and fresh stool vs. non-fresh stool) within the two groups of cats (healthy cats vs. kidney stone-diseased cats) (Table 4, Table 5, Table 6, Table 7, Table 8 and Table 9).

#### 3.3.1. Comparison of Bacterial Phyla Between Rectal Swab and Fresh Stool in Healthy Cats

In the healthy cats, the comparison of bacterial phyla between the rectal swab and fresh stool revealed statistically significant differences for the phyla Actinobacteria, Bacteroidetes, Fusobacteria, and Proteobacteria (*p* = 0.0039 for each) (Table 4). No significant difference was observed for Campylobacterota (*p* = 0.8125), Firmicutes (*p* = 0.4961), Synergistetes (*p* = 0.5), or unclassified bacteria (*p* > 0.999). No bacteria belonging to the phyla Candidatus Saccharibacteria, Chlamydiae, and Verrucomicrobia were detected with these two collection methods (Table 4).

**Table 4:** A comparison of bacterial phyla between the rectal swab (RS) and fresh stool (FS) in the healthy cats (HC). A double asterisk indicates a statistically significant difference with a *p*-value ≤ 0.01.

Phylum	Mean ± SEM for RS–HC	Mean ± SEM for FS–HC	*p*-Value	Significant
Actinobacteria	12.697 ± 6.276	31.161 ± 4.983	0.0039	Yes **
Bacteroidetes	32.681 ± 3.985	22.530 ± 4.657	0.0039	Yes **
Campylobacterota	1.757 ± 3.046	0.653 ± 0.958	0.8125	No
Candidatus Saccharibacteria	0	0	/	/
Chlamydiae	0	0	/	/
Firmicutes	42,400 ± 5257	43.877 ± 6.238	0.4961	No
Fusobacteria	5.003 ± 6.097	0.063 ± 0.046	0.0039	Yes **
Proteobacteria	5.440 ± 3.487	1.715 ± 0.65	0.0039	Yes **
Synergistetes	0.022 ± 0.051	0	0.5	No
Verrucomicrobia	0	0	/	/
Unclassified	0	0.0003 ± 0.001	>0.999	No

#### 3.3.2. Comparison of Bacterial Phyla Between Rectal Swabs and Fresh Stool in Kidney Stone-Diseased Cats

In the kidney stone-diseased cats, the comparison of bacterial phyla between the rectal swab and fresh stool showed significant differences for the phyla Actinobacteria and Proteobacteria (*p* = 0.0039) and Fusobacteria (*p* = 0.0156) (Table 5). No significant difference was observed for Bacteroidetes (*p* = 0.0547), Campylobacterota (*p* = 0.0938), Firmicutes (*p* = 0.25), Verrucomicrobia (*p* > 0.999), or unclassified bacteria (*p* = 0.25). No bacteria belonging to the phyla Candidatus Saccharibacteria, Chlamydiae, and Synergistetes were detected with these two collection methods (Table 5).

**Table 5:** A comparison of bacterial phyla between the rectal swab (RS) and fresh stool (FS) in the kidney stone-diseased cats (KSDC). A single asterisk indicates a statistically significant difference with a *p*-value < 0.05. A double asterisk indicates a statistically significant difference with a *p*-value ≤ 0.01.

Phylum	Mean ± SEM for RS–KSDC	Mean ± SEM for FS–KSDC	*p*-Value	Significant
Actinobacteria	7.314 ± 3.277	32.540 ± 10.987	0.0039	Yes **
Bacteroidetes	27.702 ± 8.415	18.887 ± 6.470	0.0547	No
Campylobacterota	3.194 ± 5.089	0.179 ± 0.289	0.0938	No
Candidatus Saccharibacteria	0	0	/	/
Chlamydiae	0	0	/	/
Firmicutes	51.528 ± 6.069	46.040 ± 11.116	0.25	No
Fusobacteria	1.431 ± 2.769	0.015 ± 0.030	0.0156	Yes *
Proteobacteria	8.830 ± 4.255	2.338 ± 3.258	0.0039	Yes **
Synergistetes	0	0	/	/
Verrucomicrobia	0.0002 ± 0.001	0	>0.999	No
Unclassified	0	0.001 ± 0.001	0.25	No

#### 3.3.3. Comparison of Bacterial Phyla Between Rectal Swab and Non-Fresh Stool in Healthy Cats

In the healthy cats, the comparison of bacterial phyla between the rectal swab and non-fresh stool (Table 6) revealed statistically significant differences for Actinobacteria and Bacteroidetes (*p* = 0.0078) and for Fusobacteria and Proteobacteria (*p* = 0.0039). No significant difference was observed for Campylobacterota (*p* = 0.25), Candidatus Saccharibacteria (*p* > 0.999), Chlamydiae (*p* = 0.5), Firmicutes (*p* = 0.8203), Synergistetes (*p* = 0.5), Verrucomicrobia (*p* > 0.999), or unclassified bacteria (*p* > 0.999) (Table 6).

**Table 6:** A comparison of bacterial phyla between the rectal swab (RS) and non-fresh stool (NFS) in the healthy cats (HC). A double asterisk indicates a statistically significant difference with a *p*-value ≤ 0.01.

Phylum	Mean ± SEM for RS–HC	Mean ± SEM for NFS–HC	*p*-Value	Significant
Actinobacteria	12.697 ± 6.276	39.430 ± 14.836	0.0078	Yes **
Bacteroidetes	32.681 ± 3.985	17.821 ± 8.717	0.0078	Yes **
Campylobacterota	1.757 ± 3.046	0.082 ± 0.110	0.25	No
Candidatus Saccharibacteria	0	0.001 ± 0.002	>0.999	No
Chlamydiae	0	0.002 ± 0.003	0.5	No
Firmicutes	42,400 ± 5257	41.180 ± 10.196	0.8203	No
Fusobacteria	5.003 ± 6.097	0.065 ± 0.078	0.0039	Yes **
Proteobacteria	5.440 ± 3.487	1.419 ± 1.399	0.0039	Yes **
Synergistetes	0.022 ± 0.051	0	0.5	No
Verrucomicrobia	0	0.0003 ± 0.001	>0.999	No
Unclassified	0	0.0003 ± 0.001	>0.999	No

#### 3.3.4. Comparison of Bacterial Phyla Between Rectal Swab and Non-Fresh Stool in Kidney Stone-Diseased Cats

In the kidney stone-diseased cats, the comparison between the rectal swab and non-fresh stool (Table 7) showed significant differences for Actinobacteria and Proteobacteria (*p* = 0.0039), for Bacteroidetes and Firmicutes (*p* = 0.0273), and for Fusobacteria (*p* = 0.0156). No significant difference was noted for Campylobacterota (*p* = 0.1563), Candidatus Saccharibacteria (*p* > 0.999), Chlamydiae (*p* > 0.999), and Verrucomicrobia (*p* > 0.999). No bacteria belonging to the phylum Synergistetes were detected with these two collection methods. The same applies to unclassified bacteria (Table 7).

**Table 7:** A comparison of bacterial phyla between the rectal swab (RS) and non-fresh stool (NFS) in the kidney stone-diseased cats (KSDC). A single asterisk indicates a statistically significant difference with a *p*-value < 0.05. A double asterisk indicates a statistically significant difference with a *p*-value ≤ 0.01.

Phylum	Mean ± SEM for RS–KSDC	Mean ± SEM for NFS–KSDC	*p*-Value	Significant
Actinobacteria	7.314 ± 3.277	39.741 ± 13.530	0.0039	Yes **
Bacteroidetes	27.702 ± 8.415	15.643 ± 9.253	0.0273	Yes *
Campylobacterota	3.194 ± 5.089	0.139 ± 0.188	0.1563	No
Candidatus Saccharibacteria	0	0.001 ± 0.003	>0.999	No
Chlamydiae	0	0.0004 ± 0.001	>0.999	No
Firmicutes	51.528 ± 6.069	43.873 ± 8.599	0.0273	Yes *
Fusobacteria	1.431 ± 2.769	0.015 ± 0.029	0.0156	Yes *
Proteobacteria	8.830 ± 4.255	0.588 ± 0.570	0.0039	Yes **
Synergistetes	0	0	/	/
Verrucomicrobia	0.0002 ± 0.001	0	>0.999	No
Unclassified	0	0	/	/

#### 3.3.5. Comparison of Bacterial Phyla Between Fresh Stool and Non-Fresh Stool in Healthy Cats

In the healthy cats, the comparison of bacterial phyla between the fresh stool and non-fresh stool (Table 8) showed significant differences only for the phylum Campylobacterota (*p* = 0.0234). No significant differences were observed for Actinobacteria (*p* = 0.0977), Bacteroidetes (*p* = 0.4258), Candidatus Saccharibacteria (*p* > 0.999), Chlamydiae (*p* = 0.5), Firmicutes (*p* = 0.4961), Fusobacteria (*p* > 0.999), Proteobacteria (*p* = 0.3008), Verrucomicrobia (*p* > 0.999), and unclassified bacteria (*p* > 0.999). No bacteria belonging to the phylum Synergistetes were detected with these two collection methods (Table 8).

**Table 8:** A comparison of bacterial phyla between the fresh stool (FS) and non-fresh stool (NFS) in the healthy cats (HC). A single asterisk indicates a statistically significant difference with a *p*-value < 0.05.

Phylum	Mean ± SEM for FS–HC	Mean ± SEM for NFS–HC	*p*-Value	Significant
Actinobacteria	31.161 ± 4.983	39.430 ± 14.836	0.0977	No
Bacteroidetes	22.530 ± 4.657	17.821 ± 8.717	0.4258	No
Campylobacterota	0.653 ± 0.958	0.082 ± 0.110	0.0234	Yes *
Candidatus Saccharibacteria	0	0.001 ± 0.002	>0.999	No
Chlamydiae	0	0.002 ± 0.003	0.5	No
Firmicutes	43.877 ± 6.238	41.180 ± 10.196	0.4961	No
Fusobacteria	0.063 ± 0.046	0.065 ± 0.078	>0.999	No
Proteobacteria	1.715 ± 0.65	1.419 ± 1.399	0.3008	No
Synergistetes	0	0	/	/
Verrucomicrobia	0	0.0003 ± 0.001	>0.999	No
Unclassified	0.0003 ± 0.001	0.0003 ± 0.001	>0.999	No

#### 3.3.6. Comparison of Bacterial Phyla Between Fresh Stool and Non-Fresh Stool in Kidney Stone-Diseased Cats

In the kidney stone-diseased cats, the comparison of bacterial phyla between the fresh stool and non-fresh stool (Table 9) showed no significant differences among the phyla. No differences were noted for Actinobacteria (*p* = 0.1641), Bacteroidetes (*p* = 0.4961), Campylobacterota (*p* = 0.8438), Candidatus Saccharibacteria (*p* > 0.999), Chlamydiae (*p* > 0.999), Firmicutes (*p* = 0.7344), Fusobacteria (*p* > 0.999), Proteobacteria (*p* = 0.3008), and unclassified bacteria (*p* = 0.25). No bacteria belonging to the phyla Synergistetes and Verrucomicrobia were detected with these two collection methods (Table 9).

**Table 9:** A comparison of bacterial phyla between the fresh stool (FS) and non-fresh stool (NFS) in the kidney stone-diseased cats (KSDC).

Phylum	Mean ± SEM for FS–KSDC	Mean ± SEM for NFS–KSDC	*p*-Value	Significant
Actinobacteria	32.540 ± 10.987	39.741 ± 13.530	0.1641	No
Bacteroidetes	18.887 ± 6.470	15.643 ± 9.253	0.4961	No
Campylobacterota	0.179 ± 0.289	0.139 ± 0.188	0.8438	No
Candidatus Saccharibacteria	0	0.001 ± 0.003	>0.999	No
Chlamydiae	0	0.0004 ± 0.001	>0.999	No
Firmicutes	46.040 ± 11.116	43.873 ± 8.599	0.7344	No
Fusobacteria	0.015 ± 0.030	0.015 ± 0.029	>0.999	No
Proteobacteria	2.338 ± 3.258	0.588 ± 0.570	0.3008	No
Synergistetes	0	0	/	/
Verrucomicrobia	0	0	/	/
Unclassified	0.001 ± 0.001	0	0.25	No

### 3.4. Comparison of Relative Abundance by Bacterial Phylum and by Bacterial Genus According to Collection Method and Studied Cat Population

**Figure 4:**
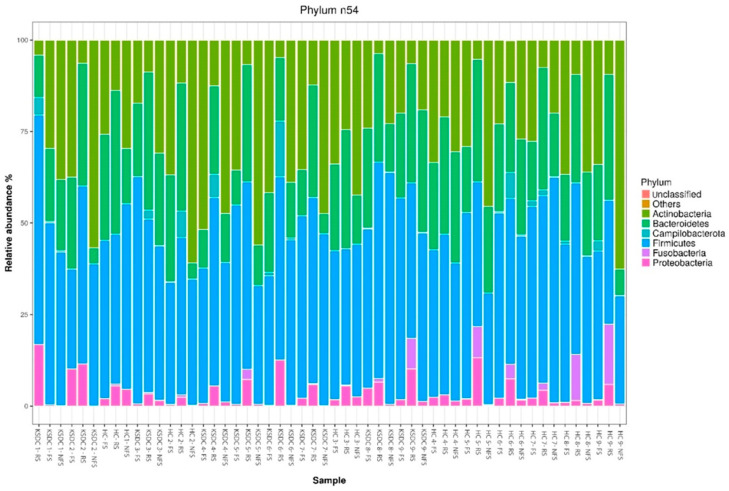
Stacked barplot graph representing the relative abundance of bacterial phyla obtained by the three stool collection methods, the rectal swab (RS), fresh stool (FS), and non-fresh stool (NFS), for the two cat populations, the healthy cats (HC) and kidney stone-diseased cats (KSDC).

**Figure 5:**
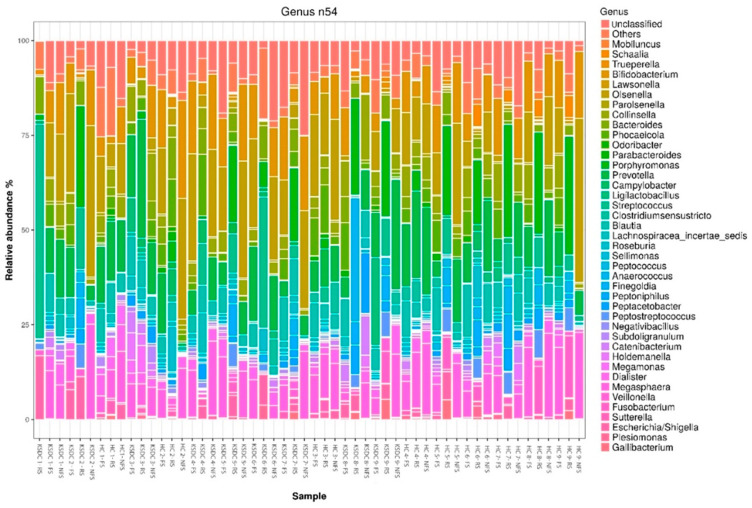
Stacked barplot graph representing the relative abundance of bacterial genera obtained by the three stool collection methods, the rectal swab (RS), fresh stool (FS), and non-fresh stool (NFS), for the two cat populations, the healthy cats (HC) and kidney stone-diseased cats (KSDC).

The relative abundances obtained for each bacterial phylum and each bacterial genus according to each collection method and for each cat are represented in Figure 4 and Figure 5, respectively.

### 3.5. Comparison of Main Alpha Diversity Indices According to Collection Method and Cat Population Studied

**Figure 6:**
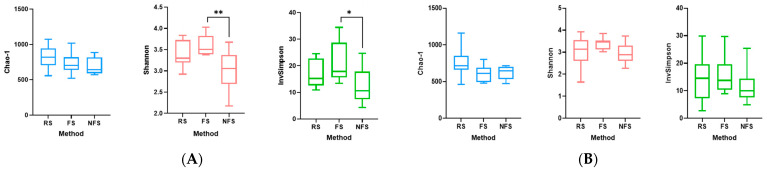
(**A**) A comparison of the Chao-1, Shannon, and InvSimpson indices in the healthy cats based on the three collection methods: the rectal swab (RS), fresh stool samples (FS), and non-fresh stool samples (NFS). Significant differences were observed when comparing the Shannon indices (*p*-value = 0.005) and InvSimpson indices (*p*-value = 0.0188) between the fresh stool and non-fresh stool samples. (**B**) A comparison of the Chao-1, Shannon, and InvSimpson indices in the kidney stone-diseased cats based on the three collection methods: the rectal swab (RS), fresh stool samples (FS), and non-fresh stool samples (NFS). No significant differences were observed for any of the indices. A single asterisk indicates a statistically significant difference with a *p*-value ≤ 0.05. A double asterisk indicates a statistically significant difference with a *p*-value ≤ 0.01.

Three of the main alpha diversity indices were calculated to assess the uniformity (Shannon diversity index), diversity (Simpson diversity index), and richness (Chao 1 diversity index). These indices were compared for each group of cats according to the three collection methods using a two-way ANOVA (Figure 6).

### 3.6. PCA of Samples Based on Cat Population Studied

#### 3.6.1. A Comparison of the PCA of the Gut Microbiota Obtained by the Rectal Swab in the Healthy Cats and the Kidney Stone-Diseased Cats

**Figure 7:**
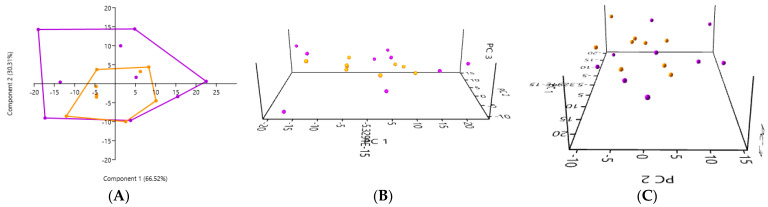
(**A**) 2D PCA graphs comparing the gut microbiota obtained by the rectal swab in the healthy cats (in orange) and cats with kidney stones (in purple). (**B**,**C**) An exploration of the same previously described patterns using 3D PCA plotting.

The PCA of the gut microbiota, performed using samples obtained by the rectal swab, did not reveal any significant differences between the healthy cats (in orange) and cats with kidney stones (in purple) (*p* = 0.6307) (Figure 7). The 2D and 3D PCA plots show an overlap between the two groups, suggesting that the composition of the gut microbiota did not present a marked difference between the healthy cats and the cats with kidney stones when the sample was collected by the rectal swab.

#### 3.6.2. A Comparison of the PCA of the Gut Microbiota in Fresh Stool Samples in the Healthy Cats and in the Kidney Stone-Diseased Cats

**Figure 8:**
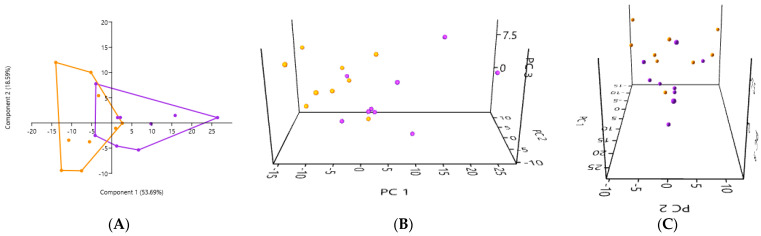
(**A**) 2D PCA graphs comparing the gut microbiota obtained from fresh stool samples in the healthy cats (in orange) and in the kidney stone-diseased cats (in purple). (**B**,**C**) An exploration of the same previously described patterns using 3D PCA plotting.

The PCA of the gut microbiota performed using the fresh stool revealed a significant difference in the composition between the healthy cats (in orange) and the cats with kidney stones (in purple) (*p* = 0.0037) (Figure 8). The 2D and 3D PCA plots only partially overlap, suggesting a difference in the composition of bacterial genera in the gut microbiota between the healthy cats and the cats with kidney stones when the sample was fresh stool.

#### 3.6.3. A Comparison of the PCA of the Gut Microbiota in Non-Fresh Stool Samples in the Healthy Cats and in the Kidney Stone-Diseased Cats

**Figure 9:**
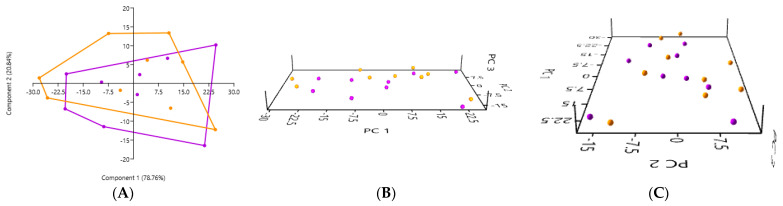
(**A**) 2D PCA graphs comparing the gut microbiota obtained from non-fresh stool samples in the healthy cats (in orange) and in the cats with kidney stones (in purple). (**B**,**C**) An exploration of the same previously described patterns using 3D PCA plotting.

The PCA of the gut microbiota performed using non-fresh stool samples did not reveal any significant difference between the healthy cats (in orange) and the cats with kidney stones (in purple) (*p* = 0.8357) (Figure 9). The 2D and 3D PCA plots show an overlap between the two groups, suggesting that the composition of the gut microbiota did not present a marked difference between the healthy cats and the cats with kidney stones when the sample was non-fresh stool.

### 3.7. A Comparison of the Mean Proportion of Bacterial Phyla According to the Population Studied

The comparison of bacterial phyla obtained by the rectal swab in the healthy cats and the kidney stone-diseased cats showed significant differences for Firmicutes (*p* = 0.0078), which was overrepresented in the kidney stone-diseased cats, as well as for Actinobacteria (*p* = 0.0273) and Fusobacteria (*p* = 0.0117), which were underrepresented (Table 10). No significant differences were observed for the other phyla.

The comparison of bacterial phyla obtained from the analysis of fresh stool samples in the healthy cats and the kidney stone-diseased cats showed a significant difference only for Fusobacteria (*p* = 0.0078), which was underrepresented in the kidney stone-diseased cats (Table 11).

The comparison of bacterial phyla obtained from the analysis of non-fresh stool samples in the healthy cats and the kidney stone-diseased cats did not show any significant difference between the two populations, regardless of the phylum studied (Table 12).

## 4. Discussion

Several veterinary studies have examined the bacterial phyla of the gut microbiota in healthy cats [33,34,35,36,37,38,39,40,41]; among them, six provided the proportion of the main phyla [33,35,36,37,38,39]. Systematic reviews have synthesized data regarding the microbiota in healthy cats [27,42,43,44,45] as well as in cats with digestive disorders [26,46].

The main bacterial phyla of the gut microbiota observed in healthy cats are similar to those observed in other mammals; they include Firmicutes, Bacteroidetes, Proteobacteria, Fusobacteria, and Actinobacteria, but the proportions vary from one study to another (Table 13).

### 4.1. Existing Sampling Techniques

Three main sampling techniques are available to collect fecal samples for the analysis of gut microbiota composition [47].

#### 4.1.1. Rectal Swab: A Preferred Method in Certain Hospital Settings

A rectal swab allows the targeted collection of bacteria adherent to the intestinal mucosa that are potentially involved in direct interactions with the mucosa, as is the case for some pathogens; these may not always be captured by stool samples [21]. A rectal swab is rapid and easy to perform and is particularly suited for detecting multidrug-resistant bacteria in a hospital setting where nosocomial infections are a concern [48,49]. The disadvantage of this method is that it limits the representation of luminal bacteria involved in metabolic functions.

#### 4.1.2. Collection of Spontaneously Produced Stool: The Standard Method

The collection of stool spontaneously produced by a patient is the most commonly used method for studying gut microbiota. It allows the collection of samples representative of bacteria present within the intestinal lumen, where bacteria involved in metabolic processes dominate. This method is non-invasive, easy to perform, and does not require the intervention of qualified medical personnel; a cat owner can simply collect the stool, which is desirable for a clinical study. However, the bacterial composition may be influenced by the duration of exposure to ambient air; this is why the present study compared freshly collected stool to that exposed to ambient air for 24 h.

#### 4.1.3. Intestinal Biopsy: A More Invasive Method

Intestinal biopsy allows the collection of mucosal samples via endoscopy. This method is primarily used in clinical settings to analyze host–microbiota interactions in inflammatory bowel diseases. Given that this method is less reproducible in clinical studies due to the anesthesia required in cats, and given that the non-invasive methods (rectal swabs and stool collection) provided sufficient information on microbiota composition, this method was not studied further.

### 4.2. Comparison of Bacterial Microbiota Based on Sampling Method

The primary objective of this preliminary study was to evaluate the impact of three sampling methods on the composition of intestinal bacterial communities in cats by comparing rectal swabs and two types of fecal samples. The results indicate that the sampling method significantly influences the detection of certain bacterial phyla, highlighting the importance of selecting a method based on the specific objectives of the study.

#### 4.2.1. Rectal Swab

The sample quantity collected by rectal swabs was always sufficient in this study.

In the two populations of cats studied—the healthy cats and cats with kidney stones—rectal swabs enabled the identification of bacterial communities distinct from those detected in fecal samples, whether freshly collected (*p* = 0.0001 for both cat populations) or exposed to ambient air for 24 h (*p* = 0.0003 in the healthy cats and *p* = 0.0001 in the kidney stone-diseased cats) (Table 1 and Table 2). This observation can be explained by the fact that rectal swabs capture a specific subset of bacteria, often more tolerant to oxygen and adherent to the intestinal mucosa.

In the healthy cats, the main phyla identified by rectal swabs were, in a descending order of frequency, Firmicutes (42.40%), Bacteroidetes (32.68%), Actinobacteria (12.70%), Proteobacteria (5.44%), and Fusobacteria (5.00%) (Table 4). Compared to samples obtained from fresh stool, the Bacteroidetes, Fusobacteria, and Proteobacteria phyla were overrepresented in samples obtained by rectal swabs (*p* = 0.0039 for each) (Table 4). Actinobacteria was underrepresented (*p* = 0.0039 for each) (Table 4), suggesting that these bacteria are primarily luminal.

In the kidney stone-diseased cats, the distribution of the different phyla remained generally similar to that observed in the healthy cats: Firmicutes (51.53%), Bacteroidetes (27.70%), Proteobacteria (8.83%), Actinobacteria (7.31%), Campylobacterota (3.19%), and Fusobacteria (1.43%). Compared to samples obtained from fresh stool, Bacteroidetes was no longer overrepresented, but as in the healthy cats, Fusobacteria and Proteobacteria were overrepresented (*p* = 0.0156 and *p* = 0.0039, respectively) and Actinobacteria was underrepresented (*p* = 0.0039) (Table 5).

The comparison of phyla proportions between the healthy cats and the cats with kidney stones shows significant differences for Firmicutes (42.40% vs. 51.53%; *p* = 0.0078), Actinobacteria (12.70% vs. 7.31%; *p* = 0.0273), and Fusobacteria (5.00% vs. 1.43%; *p* = 0.0117). These results suggest that when sampling was performed by rectal swabs, it was possible to identify dysbiosis in the kidney stone-diseased cats, characterized by an overrepresentation of Firmicutes and an underrepresentation of Actinobacteria and Fusobacteria.

#### 4.2.2. Fresh Stool

Fresh fecal samples are more representative of the luminal microbiota.

In the healthy cats, the main phyla identified in fresh stool were, in order of frequency: Firmicutes (43.88%), Actinobacteria (31.16%), Bacteroidetes (22.53%), Proteobacteria (1.72%), Campylobacterota (0.65%), and Fusobacteria (0.06%) (Table 4). Thus, in the healthy cats from this study, when the sample was fresh stool, the proportion of Actinobacteria (31.16%) was higher than the proportion of Bacteroidetes (22.53%), which contrasts with the majority of previous studies in cats where this proportion ranged from 1 to 8% [33,35,36,37,38,39] (Table 13). This observation may be due to specific characteristics of the colony studied (origins, diet, and environment).

The proportion of Actinobacteria in fresh fecal samples from the healthy cats in this colony was significantly higher than that observed in samples obtained by rectal swabs (31.16% vs. 12.70%; *p* = 0.0039). Actinobacteria, particularly some genera such as *Bifidobacterium*, are primarily luminal bacteria, colonizing the intestinal contents rather than the mucosal surface.

In the kidney stone-diseased cats, the distribution of the different phyla remained generally similar to that in the healthy cats: Firmicutes (46.04%), Actinobacteria (32.54%), Bacteroidetes (18.89%), Proteobacteria (2.34%), Campylobacterota (0.18%), and Fusobacteria (0.015%). As in the healthy cats, Actinobacteria also ranked second. Only the proportion of Fusobacteria was significantly lower in the kidney stone-diseased cats (0.063 vs. 0.015; *p* = 0.0078); when the sample was fresh stool, the proportion of Firmicutes and Actinobacteria did not differ between the healthy cats and the cats with kidney stones (*p* = 0.6523 for both phyla).

#### 4.2.3. Non-Fresh Stool

The analysis of fecal samples exposed to ambient air for 24 h did not reveal any statistically significant difference compared to the fresh fecal samples in terms of beta diversity (*p* = 0.1927 in the healthy cats and *p* = 0.3634 in the kidney stone-diseased cats), suggesting that exposure to ambient air for a period of 24 h does not substantially alter the bacterial composition of the main phyla such as Firmicutes, Bacteroidetes, Proteobacteria, and Fusobacteria. In the healthy cats, only the Campylobacterota phylum was significantly more abundant in the fresh stool compared to the non-fresh stool (0.653% vs. 0.082%; *p* = 0.0234).

In the healthy cats, the alpha diversity measured by the Shannon and Simpson indices was significantly higher in the fresh stool than in the non-fresh stool (Figure 6), reflecting a greater bacterial diversity and a more equitable distribution of communities. However, this difference was not observed in the kidney stone-diseased cats, possibly due to an initially lower biodiversity in this population.

Compared to the rectal swab samples, only the Actinobacteria phylum was more represented in the non-fresh fecal samples. Prolonged exposure to ambient air may favor the growth of aerobic Actinobacteria. Additionally, the degradation of anaerobic bacteria in non-fresh stool may accentuate this relative overrepresentation of Actinobacteria.

The lack of major alteration in the bacterial composition of the main phyla such as Firmicutes, Bacteroidetes, Proteobacteria, and Fusobacteria between the fresh and non-fresh stool—both in the healthy cats and in the kidney stone-diseased cats—is consistent with observations from other studies conducted in humans and cats showing the stability of the fecal microbiota after moderate exposure to oxygen.

In human medicine, Budding et al. showed that storage at room temperature for two hours before freezing samples obtained by rectal swabs at −20 °C maintained microbiological integrity [48]. Bassis et al. showed that the interim storage (24–48 h) of stool aliquots at temperatures likely to be available in a hospital (4 °C or −20 °C), even with one or two freeze/thaw cycles, did not significantly alter the microbiota [49]. Salonen et al. showed minimal changes in the microbiota of stool samples stored without a preservative at −20 °C or 4 °C for up to eight weeks [50].

In feline medicine, the study by Tal et al. [38], conducted on 12 healthy cats using fecal samples collected 15 min after defecation, demonstrated that the maintenance of feline fecal samples at an ambient temperature for up to four days had no effect on the bacterial composition and structure. These findings suggest a certain logistical flexibility for microbiota studies, as immediate collection after defecation is not always feasible. Thus, the analysis of fecal samples collected at home by the cat owners seemed feasible at first. However, as we will discuss later, this approach would not have been suitable for our feline study model.

Storage at −80 °C was used because this temperature was chosen for the final study, and all samples in our studies were stored in the same freezer. Storage at this temperature is considered optimal [47,51]. In addition to the previously cited studies [48,49,50,51], the study conducted by Cardona showed that a temperature of −20 °C was acceptable when the microbiota was analyzed at the level of the main phyla [52].

In the kidney stone-diseased cats, the comparison of bacterial phyla obtained from the analysis of non-fresh fecal samples showed no significant difference with the healthy cats, regardless of the phylum studied (Table 12). In contrast, a difference was observed when the sample was obtained by a rectal swab and, to a lesser extent, when the sample was fresh stool. Thus, the analysis of a non-fresh fecal sample seemed less likely to detect intestinal dysbiosis in the kidney stone-diseased cats.

Our findings are consistent with the study by Albenberg et al. [21], which demonstrated that oxygen diffuses from the intestinal tissues, creating a radial gradient where oxygen-tolerant organisms, notably from the phyla Proteobacteria and Actinobacteria, increase near the mucosa, whereas anaerobic bacteria, such as those from the phyla Firmicutes and Bacteroidetes, predominate in fecal matter.

Similarly, in alignment with our study, the research by Jones et al. [24] comparing intestinal microbial communities across three sample types—stool, rectal swabs, and mucosal biopsies—showed that fecal samples primarily reflect luminal microbiota, whereas rectal swabs capture both luminal and mucosal microbiota. In their study, the bacterial communities in the rectal swabs were closer to those in the fecal samples than in the rectal biopsies, with the swabs presenting a higher abundance of aerobic bacteria due to the decreasing oxygen gradient from the mucus layer toward the intestinal lumen. Interindividual differences were more significant than the differences attributable to the sample type.

Our observations also align with the recent study by Nowicki et al. [23] conducted on women with breast cancer. This study showed that colonic biopsy samples captured taxa specific to the mucosa, whereas stool samples, whether collected at home or during a sigmoidoscopy, exhibited greater diversity and a better representation of common taxa. The authors suggested that biopsies and stool samples, as they provide complementary information, should be used together in studies of the intestinal microbiome.

In contrast, our results differ from those of other studies in human medicine that have reported very similar bacterial communities between rectal swab samples and fecal samples within the same individual [25,48,49].

### 4.3. Rarity of Certain Phyla

The phyla Campylobacterota, Candidatus Saccharibacteria, Chlamydiae, Synergistetes, and Verrucomicrobia showed very low or even undetectable abundances regardless of the sampling method, indicating that they are only weakly represented in these bacterial populations, independent of the collection method. Similarly, for unclassified bacteria, the abundance was very low regardless of the collection method. Low or undetectable abundances could reflect limitations in current taxonomic classification or difficulties in detecting certain groups with the techniques used.

These findings emphasize the importance of method selection and storage conditions for accurately characterizing the intestinal microbiota, particularly when identifying less common phyla or investigating specific microbial alterations.

### 4.4. Comparison of Bacterial Microbiota Based on Studied Population

#### 4.4.1. Comparison of PCA Graphs

The PCA results suggest that the sampling method influenced the ability to detect differences in the composition of the intestinal microbiota between the healthy cats and cats with kidney stones.

The PCA of the samples obtained via rectal swabs did not show a significant difference in the microbiota composition between the healthy cats and the cats with kidney stones (*p* = 0.6307). This can be attributed to the fact that rectal swabs mainly collect bacteria adhering to the intestinal mucosa, which are more tolerant to oxygen and potentially less involved in metabolic functions.

In contrast, the PCA of the fresh fecal samples revealed a significant difference in the bacterial composition between the healthy cats and the cats with kidney stones (*p* = 0.0037). This might be related to the fact that fresh stool contains luminal microbiota, which is more involved in metabolic functions. The dysbiosis detected here could reflect an alteration in the luminal bacteria responsible for oxalate degradation, suggesting that the luminal microbiota may play a role in the pathogenesis of feline calcium oxalate urolithiasis.

The PCA of the non-fresh fecal samples did not show a significant difference in the microbiota composition between the healthy cats and the cats with kidney stones (*p* = 0.8357). Given the previous observations, this suggests that exposure to ambient air for 24 h may have eliminated strictly anaerobic bacteria involved in the pathogenesis of feline urolithiasis, limiting the ability to identify differences in the intestinal microbiota composition between the two cat populations.

#### 4.4.2. Comparison of Bacterial Phyla

A comparative analysis of bacterial phyla identified in published studies and our study reveals similarities as well as notable differences, which can be partly explained by variations in the diet, lifestyle, sampling methods, storage conditions, and populations studied.

##### Firmicutes

In studies conducted on cats, Firmicutes is consistently the dominant phylum, representing approximately 60% of the microbial community (Table 13). This predominance of Firmicutes is common in mammals. Our results confirm this dominance, with proportions close to 42% in the healthy cats, regardless of the collection method. This high representation of Firmicutes is consistent with their major role in fermenting fibers into short-chain fatty acids (SCFAs).

In our study, the proportion of Firmicutes did not differ statistically between the healthy cats and the cats with kidney stones when the sample analyzed was a fresh fecal sample (43.88% vs. 46.04%; *p* = 0.6523) or a non-fresh fecal sample (41.18% vs. 43.87%; *p* = 0.3008). However, the proportion of Firmicutes was higher in the kidney stone-diseased cats when the sample was obtained via a rectal swab (42.40% vs. 51.53%; *p* = 0.0078). This apparent overrepresentation of Firmicutes is counterintuitive and could be explained by the decrease in other phyla (Bacteroidetes, Fusobacteria, and Actinobacteria) when the sampling method used was a rectal swab.

##### Bacteroidetes

Bacteroidetes is the second most represented phylum in all studies, with percentages varying from 10% to 20% (Table 13). This phylum is involved in polysaccharide degradation, producing SCFAs.

Our results show high proportions of Bacteroidetes in the healthy cats in samples obtained via rectal swabs (32.68%), lower proportions in samples obtained from fresh stool (22.53%), and even lower proportions in samples obtained from non-fresh stool (17.82%). This trend was also observed in the kidney stone-diseased cats. Bacteroidetes were overrepresented in samples obtained via rectal swabs compared to samples obtained from stool.

There was no significant difference in the proportion of Bacteroidetes between the healthy cats and the cats with kidney stones, regardless of the sampling method used.

##### Proteobacteria

The phylum Proteobacteria represents 5 to 15% of the bacterial community in published studies (Table 13). In our study, the proportion of Proteobacteria was higher in samples obtained via rectal swabs (5.44%) compared to fresh stool (1.72%) and non-fresh stool (1.42%). A similar trend was observed in the kidney stone-diseased cats, with proportions of 8.83%, 2.34%, and 0.59%, respectively.

No significant difference was observed in the proportions of Proteobacteria between the healthy cats and the cats with kidney stones, regardless of the sampling method used.

##### Fusobacteria

The phylum Fusobacteria is present in proportions ranging from 1 to 5% in the intestinal microbiota (Table 13). This phylum is involved in protein and amino acid degradation, which produces short-chain fatty acids like butyrate.

In the healthy cats, Fusobacteria were overrepresented in samples obtained via rectal swabs (5.00%) compared to samples obtained from fresh stool (0.063%; *p* = 0.0039) or non-fresh stool (0.065%; *p* = 0.0039).

In the kidney stone-diseased cats, Fusobacteria were also overrepresented in samples obtained via rectal swabs (1.43%) compared to samples obtained from fresh stool (0.015%; *p* = 0.0156). This observation is counterintuitive since this phylum mainly includes strictly anaerobic bacteria, whose proportions are typically reduced in the presence of oxygen.

In the kidney stone-diseased cats, Fusobacteria were underrepresented, compared to those in the healthy cats, whether the sample was obtained by a rectal swab or from fresh stool. This difference was no longer observed in non-fresh fecal samples. This finding is also counterintuitive; the degradation of other oxygen-sensitive bacterial phyla altering the overall composition of the microbiota could mask the initial difference in Fusobacteria observed in rectal swabs or fresh fecal samples. Thus, exposure to ambient air resulted in a loss of information, making it impossible to identify dysbiosis in this study model.

##### Actinobacteria

The phylum Actinobacteria is generally a minor component of the intestinal microbiota, with proportions ranging from 1% to 5% in all studies. However, this phylum is important for health due to the role of certain genera like *Bifidobacterium*, which contribute to vitamin production and inhibit pathogen growth.

In the healthy cats, non-fresh fecal samples showed a slight increase in this phylum (39.43%) compared to the fresh stool (31.16%), which can be attributed to oxygen exposure favoring the growth of certain aerobic species; however, this difference was not statistically significant. This phenomenon was also observed in the study by Tal et al. [38].

In the healthy cats, the relatively lower presence of Actinobacteria in the rectal swabs (12.70%) compared to the fresh stool (31.16%) (*p* = 0.0039) is counterintuitive and could possibly be explained by competition from other aerobic bacteria.

#### 4.4.3. Summary of Bacterial Microbiota Comparison

This study provides seemingly conflicting results between the PCA comparison of samples and the comparison of bacterial phylum proportions. The PCA comparison revealed a significant difference in the bacterial composition between the healthy cats and the cats with kidney stones only when the sample was a fresh fecal sample (*p* = 0.0037). This finding suggests that fresh stool should be favored for the study of dysbiosis associated with feline nephrolithiasis. However, a difference in the proportion of phyla between the healthy cats and the kidney stone-diseased cats was observed for Firmicutes, Actinobacteria, and Fusobacteria only when the sample was obtained via a rectal swab. This suggests that rectal swabs may have been more likely to identify dysbiosis in the kidney stone-diseased cats.

The divergence between these two approaches can be explained by their respective objectives; the PCA aimed to detect global variations in the microbiota composition between the two populations of cats, whereas the comparative analysis of bacterial phyla focused on differences in the proportions of the main bacterial phyla. The overrepresentation of Firmicutes in samples obtained by rectal swabs in the kidney stone-diseased cats may reflect the relative scarcity of other bacterial phyla (Bacteroidetes, Fusobacteria, and Actinobacteria). This dilution effect makes variations in phylum proportions more apparent, even if they do not result in a sufficiently significant global compositional difference to be detected by PCA. The apparent discrepancy between these methods underscores their complementarity.

The differences observed between the healthy cats and the cats with kidney stones in this preliminary study suggest a potential link between intestinal dysbiosis and kidney stone formation in cats. The confirmation of the involvement of the intestinal microbiota in the pathogenesis of renal lithiasis in cats opens new avenues for research focused on correcting dysbiosis, for instance, through dietary changes, targeted antibiotic therapy, prebiotics, probiotics [53,54], and even fecal microbiota transplantation [55,56].

### 4.5. Advantages of This Animal Experimental Model

The 18 cats studied demonstrate the benefits of an animal experimental model, as the individuals studied were of the same breed, were sexually neutral from a young age, were of the same age (about 9 years), came from the same colony, had the same medical history, lived in the same environment (for 9 years), all received the same standard diet every day, and had never been given antibiotics. The cats in this study were not in renal failure, so the factors of microbiota variation associated with renal failure [57,58] were not taken into account in this study.

Although there were two male cats in the group of healthy cats and sixteen females distributed between the two groups, none of the cats in the study have ever experienced hormonal influence as all the cats were sterilized before puberty. Therefore, the composition of their microbiota had not been influenced by the presence of sex hormones. Anatomical differences between male and female cats only concern the lower urinary tract, not the upper urinary tract. Several studies conducted in mice and in humans have not shown an influence of sex on microbiota composition [59]. In the studies conducted in humans with kidney stones, no distinction was made between men and women; our cats have the advantage of all having the same sexual status, in that they have all been sexually neutered since birth.

The exact composition of the kidney stones was identified only for three of the nine cats with lithiasis, which had died. As expected, their stones were composed entirely of calcium oxalate monohydrate. For the remaining six cats, the calcium oxalate composition of their stones was inferred given that 98% of upper urinary tract stones in cats are composed of calcium oxalate [9,10]. Struvite stones, by contrast, often have distinct radiographic characteristics: they are less radiodense, larger, smoother, and sometimes pyramid-shaped. Therefore, the likelihood of non-calcium oxalate stones among the remaining six cats was minimal. In human studies, some have failed to distinguish between stone types [8,60], despite calcium oxalate stones constituting only 80% of kidney stones in humans. In veterinary studies, dogs with calcium oxalate stones were recruited from pet owners, resulting in significant variability in their diet and environmental factors [61,62,63]. Similarly, eight prior studies in humans did not consider for variables such as diet, lifestyle, underlying diseases, medications, and geographic region, which significantly influence microbiota composition [64]. The ability to control environmental factors in cats raised under experimental conditions makes them an ideal model for studying human urolithiasis.

### 4.6. Study Limitations and Potential Research Directions

One limitation of this preliminary study lies in its focus on examining the main phyla present in the collected samples, without delving deeper into a lower-taxonomic-level analysis. This gap was partially addressed by a subsequent microbiota analysis conducted at the species level in the same 18 cats as part of the main study [16]. In the preliminary study, which examined the intestinal microbiota at the phylum level using fresh fecal samples, only alterations related to the phylum Fusobacteria were observed in the kidney stone-diseased cats, whereas in the main study, an in-depth species-level analysis identified a greater number of differences in the microbiota composition between cats with renal calculi and healthy cats.

A limitation of both studies conducted on this feline model is that they did not explore the metabolic functions of the microbiota. To move beyond mere taxonomic characterization, functional analyses such as functional metagenomics, metatranscriptomics, metabolomics, metaproteomics, or even short-chain fatty acid (SCFA) analysis would be necessary. These approaches would help determine whether certain metabolic pathways are disrupted in the kidney stone-diseased cats, which could provide new avenues for the prevention and treatment of renal calculi.

## 5. Conclusions

This study shows that, within the same individual, the method of fecal sample collection influences the composition of the detected bacterial communities, particularly for the main phyla; notable differences exist between the rectal swabs and fecal samples, whether fresh or not. Thus, the collection method alone can alter the outcomes of a study.

When comparing the two populations of cats—healthy cats and cats with kidney stones—PCA revealed a difference in the gut microbiota composition only when fresh stool samples were used. This suggests that the intestinal bacteria involved in the pathogenesis of feline kidney stones are luminal and strictly anaerobic bacteria. Consequently, exposure to ambient air resulted in a loss of information, preventing the identification of dysbiosis. For clinical studies, non-fresh stool samples provided by owners does not appear suitable for studying the gut microbiota of cats with kidney stones; fresh stool should be favored.

Paradoxically, when comparing the two populations of cats, only the rectal swabs revealed a difference in the proportion of major phyla.

These observations suggest that, for a deeper understanding of the role of the gut microbiota in the formation of kidney stones in cats, it would be beneficial to use both rectal swabs (performed on an anesthetized cat) and fresh stool samples. Ideally, future studies should incorporate bacterial identification at the species level and explore the metabolic functions of both the gut and urinary microbiota.

## Figures and Tables

**Table 1:** *p*-values associated with the comparisons of different collection methods. A triple asterisk indicates a statistically significant difference with a *p*-value ≤ 0.001.

	Rectal Swab	Fresh Stool	Non-Fresh Stool
Rectal swab	-	0.0001 ***	0.0003 ***
Fresh stool	0.0001 ***	-	0.1927
Non-fresh stool	0.0003 ***	0.1927	-

**Table 2:** *p*-values associated with the comparisons of different collection methods. A triple asterisk indicates a statistically significant difference with a *p*-value ≤ 0.001.

	Rectal Swab	Fresh Stool	Non-Fresh Stool
Rectal swab	-	0.0001 ***	0.0001 ***
Fresh stool	0.0001 ***	-	0.3634
Non-fresh stool	0.0001 ***	0.3634	-

**Table 3:** *p*-values associated with the comparisons of different collection methods. A triple asterisk indicates a statistically significant difference with a *p*-value ≤ 0.001.

	Rectal Swab	Fresh Stool	Non-Fresh Stool
Rectal swab	-	0.0003 ***	0.0003 ***
Fresh stool	0.0003 ***	-	0.0651
Non-fresh stool	0.0003 ***	0.0651	-

**Table 10:** Comparison of bacterial phyla obtained by the rectal swab (RS) in the healthy cats (HC) and the kidney stone-diseased cats (KSDC). A single asterisk indicates a statistically significant difference with a *p*-value < 0.05. A double asterisk indicates a statistically significant difference with a *p*-value ≤ 0.01.

Phylum	Mean ± SEM for RS–HC	Mean ± SEM for RS–KSDC	*p*-Value	Significant
Actinobacteria	12.697 ± 6.276	7.314 ± 3.277	0.0273	Yes *
Bacteroidetes	32.681 ± 3.985	27.702 ± 8.415	0.0742	No
Campylobacterota	1.757 ± 3.046	3.194 ± 5.089	0.6523	No
Candidatus Saccharibacteria	0	0	/	/
Chlamydiae	0	0	/	/
Firmicutes	42,400 ± 5257	51.528 ± 6.069	0.0078	Yes **
Fusobacteria	5.003 ± 6.097	1.431 ± 2.769	0.0117	Yes *
Proteobacteria	5.440 ± 3.487	8.830 ± 4.255	0.1289	No
Synergistetes	0.022 ± 0.051	0	0.5	No
Verrucomicrobia	0	0.0002 ± 0.001	1	No
Unclassified	0	0	/	/

**Table 11:** A comparison of bacterial phyla obtained from the analysis of fresh stool samples (FS) in the healthy cats (HC) and the kidney stone-diseased cats (KSDC). A double asterisk indicates a statistically significant difference with a *p*-value ≤ 0.01.

Phylum	Mean ± SEM for FS–HC	Mean ± SEM for FS–KSDC	*p*-Value	Significant
Actinobacteria	31.161 ± 4.983	32.540 ± 10.987	0.6523	No
Bacteroidetes	22.530 ± 4.657	18.887 ± 6.470	0.1641	No
Campylobacterota	0.653 ± 0.958	0.179 ± 0.289	0.4961	No
Candidatus Saccharibacteria	0	0	/	/
Chlamydiae	0	0	/	/
Firmicutes	43.877 ± 6.238	46.040 ± 11.116	0.6523	No
Fusobacteria	0.063 ± 0.046	0.015 ± 0.030	0.0078	Yes **
Proteobacteria	1.715 ± 0.65	2.338 ± 3.258	0.7344	No
Synergistetes	0	0	/	/
Verrucomicrobia	0	0	/	/
Unclassified	0.0003 ± 0.001	0.001 ± 0.001	0.25	No

**Table 12:** A comparison of bacterial phyla obtained from the analysis of non-fresh stool samples (NFS) in the healthy cats (HC) and the kidney stone-diseased cats (KSDC).

Phylum	Mean ± SEM for NFS–HC	Mean ± SEM for NFS–KSDC	*p*-Value	Significant
Actinobacteria	39.430 ± 14.836	39.741 ± 13.530	0.8203	No
Bacteroidetes	17.821 ± 8.717	15.643 ± 9.253	0.5703	No
Campylobacterota	0.082 ± 0.110	0.139 ± 0.188	0.7422	No
Candidatus Saccharibacteria	0.001 ± 0.002	0.001 ± 0.003	1	No
Chlamydiae	0.002 ± 0.003	0.0004 ± 0.001	0.5	No
Firmicutes	41.180 ± 10.196	43.873 ± 8.599	0.3008	No
Fusobacteria	0.065 ± 0.078	0.015 ± 0.029	0.2969	No
Proteobacteria	1.419 ± 1.399	0.588 ± 0.570	0.1289	No
Synergistetes	0	0	/	/
Verrucomicrobia	0.0003 ± 0.001	0	1	No
Unclassified	0.0003 ± 0.001	0	1	No

**Table 13:** A comparison of the proportion of main phyla in various published studies and in the present study.

Phylum	Ritchie et al. (2008) [33] (Luminal Intestinal Content)	Handl et al. (2011) [35] (Stool)	Barry et al. (2012) [36] (Stool)	Tun et al. (2012) [37] (Stool)	Tal et al. (2017) [38] (Fresh and Non-Fresh Stool)	Jha et al. (2020) [39] (Stool)	Present Study, Joubran et al. (2024) (Rectal Swab)		Present Study, Joubran et al. (2024) (Fresh Stool)		Present Study, Joubran et al. (2024) (Non-Fresh Stool)	
	5 healthy cats	12 healthy cats	4 healthy cats	5 healthy cats	12 healthy cats	46 healthy cats	9 healthy cats	9 cats with kidney stones	9 healthy cats	9 cats with kidney stones	9 healthy cats	9 cats with kidney stones
Firmicutes	68%	92.1%	36.3%	12.98%	~70–80%	38.43%	42.4%	51.53%	43.88%	46.04%	41.18%	43.87%
Bacteroidetes	10%	0.45%	36.1%	67.54%	~3–5%	14.30%	32.68%	27.7%	22.53%	18.89%	17.82%	15.64%
Proteobacteria	14%	unspecified	12.4%	5.85%	~5–10%	37.47%	5.44%	8.83%	1.72%	2.34%	1.42%	0.59%
Fusobacteria	5%	0.04%	-	0.68%	<1%	1.58%	5%	1.43%	0.063%	0.015%	0.065%	0.015%
Actinobacteria	4%	7.31%	7.7%	1.16%	~10–15%	8.06%	12.7%	7.31%	31.16%	32.54%	39.43%	39.74%

## Data Availability

The original contributions presented in the study are included in the article/Supplementary Material, further inquiries can be directed to the corresponding author.

## Supplementary Materials

The composition of the food can be found on the following website: https://www.royalcanin.com/au/cats/products/vet-products/mature-consult-2724 (accessed on 13 October 2024).
